# Late‐onset laryngeal paralysis: Owner perception of quality of life and cause of death

**DOI:** 10.1002/vms3.240

**Published:** 2020-01-25

**Authors:** Susannah J. Sample, Allison Stilin, Emily E. Binversie, Lauren A. Baker, Robert J. Hardie

**Affiliations:** ^1^ Department of Surgical Sciences School of Veterinary Medicine University of Wisconsin‐Madison Madison WI USA

**Keywords:** arytenoid lateralization, Labrador Retriever, laryngeal paralysis, polyneuropathy, quality of life

## Abstract

**Background:**

Late‐onset laryngeal paralysis (LoLP) is an idiopathic disease of older dogs, and is common in the Labrador Retriever. Owner perspective of how LoLP affects their pet's quality of life (QOL), the degree to which LoLP is perceived to be a life‐limiting disease, and how a glottic opening procedure affects these perceptions is not known.

**Objectives:**

(a) To determine owner's perception of late‐onset laryngeal paralysis (LoLP) with respect to their dog's QOL; (b) To determine whether LoLP is considered by owners to be a life‐limiting disease; (c) To evaluate whether a glottic opening procedure altered QOL and perceived cause of death in affected dogs.

**Methods:**

Owners of Labrador Retrievers with LoLP completed a questionnaire. Questions were asked pertaining to a dog's LoLP, including clinical progression and perception of cause of death, and whether a glottic opening procedure was undertaken. Owners also completed a pet‐owner administered QOL survey.

**Results:**

Seventy‐six owners participated. Overall, 94% of owners felt their dog's LoLP affected QOL, and 47% of owners felt LoLP was a large contributing factor in their dog's death. Dogs that underwent a glottic opening procedure were reported to have a better QOL, and the contribution of LoLP towards their death was less than dogs that did not have surgery.

**Conclusion:**

Owners of Labrador Retrievers with LoLP perceive LoLP to be a life‐limiting disease that negatively impacts their dog's QOL. Arytenoid lateralization surgery had a positive impact on QOL in affected dogs.

## Graphical abstract

Owner perspective of how late‐onset laryngeal paralysis (LoLP) affects their pet's quality of life, the degree to which LoLP is perceived to be a life limiting disease, and how a glottic opening procedure affects these perceptions is not known. Seventy six owners of dogs affected with LoLP were surveyed. Results show that LoLP is considered by owners to be a life‐limiting disease that negatively impacts their dog's quality of life, and arytenoid lateralization surgery had a positive impact on quality of life for affected dogs.

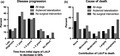

## 1 INTRODUCTION

Late‐onset laryngeal paralysis (LoLP) is a common idiopathic degenerative neurological disease of older dogs. LoLP is often associated with a polyneuropathy syndrome (Jeffery, Talbot, Smith, & Bacon, 2006; Stanley, Hauptman, Fritz, Rosenstein, & Kinns, 2010), with clinical signs including upper respiratory obstruction, pelvic limb weakness and evidence of oesophageal dysmotility (Jeffery et al., 2006; Stanley et al., 2010). About 70% of LoLP cases occur in the Labrador Retriever (Jeffery et al., 2006; Thieman, Krahwinkel, Sims, & Shelton, 2010). LoLP typically develops by 11 years of age (Granger, 2011). The condition appears to be a peripheral neuropathy, with degenerative changes present in peripheral nerves (Braund, Steinberg, & Shores, 1989; Thieman et al., 2010); it remains unknown whether the disease also affects the central nervous system. The underlying pathogenesis of LoLP is not understood. It is currently unclear whether LoLP is due to a single‐pathological mechanism or is a common clinical phenotype resulting from a variety of underlying pathologies, as is the case with many human neurodegenerative diseases (Li, 2012).

Clinical signs of LoLP are often the result of a degenerative process affecting peripheral motor nerves (Braund et al., 1989; Stanley et al., 2010; Thieman et al., 2010). Similar to many human peripheral neuropathies, LoLP appears to be commonly due to a length‐dependent neuropathy, wherein nerves with the longest axons are most severely affected (Mackin, 1994). Some of the longest peripheral motor nerves in the dog include the recurrent laryngeal nerve and the sciatic nerve with its associated branches. Degeneration of the recurrent laryngeal nerve results in laryngeal paralysis with subsequent upper respiratory obstruction resulting in dyspnoea and exercise intolerance, and is also likely responsible for the development of clinical signs associated with oesophageal dysmotility (Stanley et al., 2010; Tarvin, Twedt, & Monnet, 2016). Similarly, dogs with LoLP often develop paraparesis and decreased withdrawal reflexes in their pelvic limbs as a result of degeneration of hindlimb innervation (Gaber, Amis, & LeCouteur, 1985; Jeffery et al., 2006; Stanley et al., 2010).

Quality of life (QOL) and owner perception is an important topic in companion animal medicine. Owner perception of how LoLP affects their dog's QOL, and the degree to which LoLP is perceived to contribute to death, has not been documented. Furthermore, how glottic opening procedures impact QOL and whether these procedures result in a difference in perceived cause of death remains unknown. A variety of methods have been developed and validated to access QOL in dogs (Budke et al., 2008; Lavan, 2013; Reid, Wiseman‐Orr, Scott, & Nolan, 2013). The aims of this study were: (a) To determine owner's perception of LoLP with respect to their dog's QOL; (b) To determine whether LoLP is considered by owners to be a life‐limiting disease; (c) To evaluate whether a glottic opening procedure altered QOL and perceived cause of death in affected dogs. We used an owner questionnaire, which included a QOL survey, to evaluate a population of Labrador Retriever dogs diagnosed with LoLP. We hypothesized that owners of affected Labrador Retrievers perceive the condition to be a life‐limiting disease that negatively affects their dog's QOL.

## 2 MATERIALS AND METHODS

### 2.1 Case selection criteria

Owners of Labrador Retrievers with LoLP were recruited between July 2017 and May 2018 to participate in this study. Dogs identified for this study were previously or concurrently enrolled in an ongoing LoLP genetics study undertaken at the author's institution (Sample et al., 2017), for which enrolment occurred between May 2014 and May 2018. Recruitment occurred throughout the United States and Canada. Dogs were diagnosed with LoLP through a combination of physical examination, history, neurological examination and airway examination. For inclusion in this study, dogs had to have, as a minimum, diagnosis of LoLP by a veterinarian, severely increased upper airway noise that was most evident after exercise, and had to be at least 7 years of age before onset of clinical signs. For dogs evaluated at our institution, in addition to the minimum criteria listed above, a neurological examination performed by a board‐certified veterinary neurologist or surgeon experienced with neurological examination. Dogs were excluded if they were diabetic, had a history of chemotherapy or uncontrolled endocrinopathy, or if evaluation or history indicated a cause of laryngeal paralysis other than idiopathic (Sample et al., 2017).

### 2.2 Questionnaire

The questionnaire (Figure S1) contained two parts. The first part was comprised of a series of questions pertaining to the dog's LoLP condition. These included gender, neuter status, the age at which the dog was noted to have developed clinical signs of LoLP, whether the dog was still alive, whether the dog had surgery to relieve airway obstruction and if so, whether the owners felt surgery positively influenced their dog's QOL. Follow‐up regarding the type of glottic opening surgery performed was obtained after the questionnaires were completed. Owners were also asked to indicate how quickly and if, from when they first noticed clinical signs of LoLP, they felt LoLP progressed to the point where their dog's QOL was affected (Figure S1). These responses were scored from 1 to 4, such that a higher score was associated with a longer time between when owners first noticed signs of LoLP and when they felt their dog's QOL was affected due to the disease. An option of “I didn't know my dog had laryngeal paralysis until he/she was very affected” was included; for analysis, these responses were considered absent values, and the number of owners with this response was recorded. Owners were also asked about the extent they felt LoLP contributed to their dog's death or their decision to perform humane euthanasia (Figure S1). These responses were scored from 1 to 5, such that a higher score indicated that owners felt LoLP was more associated with their dog's cause of death.

The second part of the questionnaire was comprised of the pet‐owner administered, multi‐domain canine health‐related QOL survey (CHQLS), that has previously been validated for healthy dogs (Lavan, 2013). This survey divides a total of 15 questions into four lifestyle and QOL factors, including three questions related to happiness, five questions related to physical functioning, three questions related to hygiene and three questions related to mental status, the sum of which results in an overall QOL score (total CHQLS score). Each question is scored from 1 to 3, resulting in a maximum score of 9 for happiness, 15 for physical functioning, 9 for hygiene, 9 for mental status and thus a total of 45 for the CHQLS QOL score.

Two additional items were embedded within the CHQLS that focused specifically on issues associated with LoLP, which were as follows: “My pet is able to undertake activities he/she wants” and “My pet's breathing prevents activity”. As with the CHQLS, owners were asked to respond to these statements by circling whether they agreed, were neutral, or disagreed. These two items were analysed separately from the CHQLS results.

Owners were instructed to answer questions relative to their dog's current status, if still alive, or their dog's status within the 6 months before death. Owners were also asked to list additional known disease(s) and had the opportunity to add other comments or information they felt were important. Owners were contacted by e‐mail, phone or in person to participate, and were able to do so through an online questionnaire, by hand, or via telephone, depending on participant preference. Whether owners completed the questionnaire through a direct conversation or in private was recorded.

### 2.3 Statistical analysis

Data were analysed for normality using the D’Agostino & Pearson normality test. To determine if the method by which the questionnaire was completed influenced results, whether in private or through a direct conversation, the Spearman rank test was used. To compare owner scoring of questionnaire items between dogs that had glottic opening surgery and those that did not, the Mann–Whitney test or Student's *t*‐test was used, as appropriate. Internal consistency for the questionnaire was assessed using the Cronbach α coefficient (CAC), which ranges from 0 to 1. The higher the CAC, the higher the evidence that items reliably represent a particular owner perception. Low CAC indicates either insufficient questions or that questions have little in common. A CAC score >0.7 indicates good internal consistency. A CAC was determined for the questions associated with the CHQLS survey. Results were considered significant at *p < *.05. Data were reported as mean ± standard deviation for parametric data and median (range) for non‐parametric data.

## 3 RESULTS

A total of 130 dog owners were identified as qualifying for this study and contacted for study participation. Of these, 36 did not respond to the e‐mail or phone request, 11 were not able to be contacted due to a lack of current contact information, two declined to participate and five did not respond to all survey questions. Seventy‐six owners participated in this study. Fifty‐five (72%) dogs were diagnosed with LoLP at our institutional practice, nine (12%) were diagnosed with LoLP and had an airway opening surgery at another referral practice, and 12 (16%) were diagnosed through their primary care veterinarian. All dogs enrolled were reported to have evidence of hindlimb weakness; all 55 dogs evaluated at our institutional practice had hindlimb paraparesis and decreased withdrawal reflexes. Forty‐one dogs (54%) were reported to be neutered males, 27 (36%) spayed females, 5 (7%) intact males and 3 (4%) intact females. Thirty‐three dogs (43%) had a glottic opening surgery and 43 dogs (57%) did not. All dogs who had a glottic opening procedure were reported to have underwent unilateral arytenoid lateralization. At the time of the survey, 42 dogs (55%) were reported to be dead and 34 dogs (45%) were alive. The median time between time of death and survey completion was 0.96 years (range 0–4.51 years).

The CAC value for the questionnaire was 0.73. This indicates good reliability. Sixty‐four owners responded to the questionnaire online or through the mail, and 12 responded over the telephone. There was no correlation between any of the questionnaire responses and the means by which owners completed the questionnaire (Bowling, 2005).

The mean age that owners first noted signs of LoLP was 10.3 ± 1.4 years. The mean age of dogs that had unilateral arytenoid lateralization were significantly younger (*p* = .03) at the time owners first noticed signs of LoLP than dogs who did not have surgery (9.9 ± 1.3, 10.6 ± 1.5 years, respectively) (Table 1).

**Table 1:** Differences in responses between owners of dogs who had a unilateral arytenoid lateralization and dogs that did not have an airway opening procedure

Variable	All dogs	Unilateral arytenoid lateralization	No surgical intervention	*p*‐value
Age of onset (years)	10.3 ± 1.4	9.9 ± 1.3	10.6 ± 1.5*	.03
Age of death (years)	13.2 ± 1.6	13.6 ± 1.6	12.9 ± 1.7	.16
Disease progression	3 (2–5)	3 (2–4)	3 (2–5)	.25
Contribution of LOLP to death	2 (1–5)	1.5 (1–5)	4 (1–5)**	.007
Total CHQLS score	37 (20–45)	38.21 ± 3.8	35.21 ± 5.6**	.001

Note

Data are reported as mean ± standard deviation or median (range), as appropriate. QOL, quality of life; CHQLS, canine health‐related QOL survey. *n* = 20–76 responses. *p < .05, **p < .01 versus dogs that had unilateral arytenoid lateralization surgery.

The mean age of death for the 42 dogs reported to be deceased at the time of survey completion was 13.2 ± 1.6 years. The mean age of death was not statistically different between dogs that had unilateral arytenoid lateralization surgery (*n* = 20, 13.6 ± 1.6) and those that did not (*n* = 22, 12.9 ± 1.7) (*p* = .16) (Table 1). The median time between owner reported age of onset and death was 2.6 years (range 0–9.7 years).

**Figure 1:**
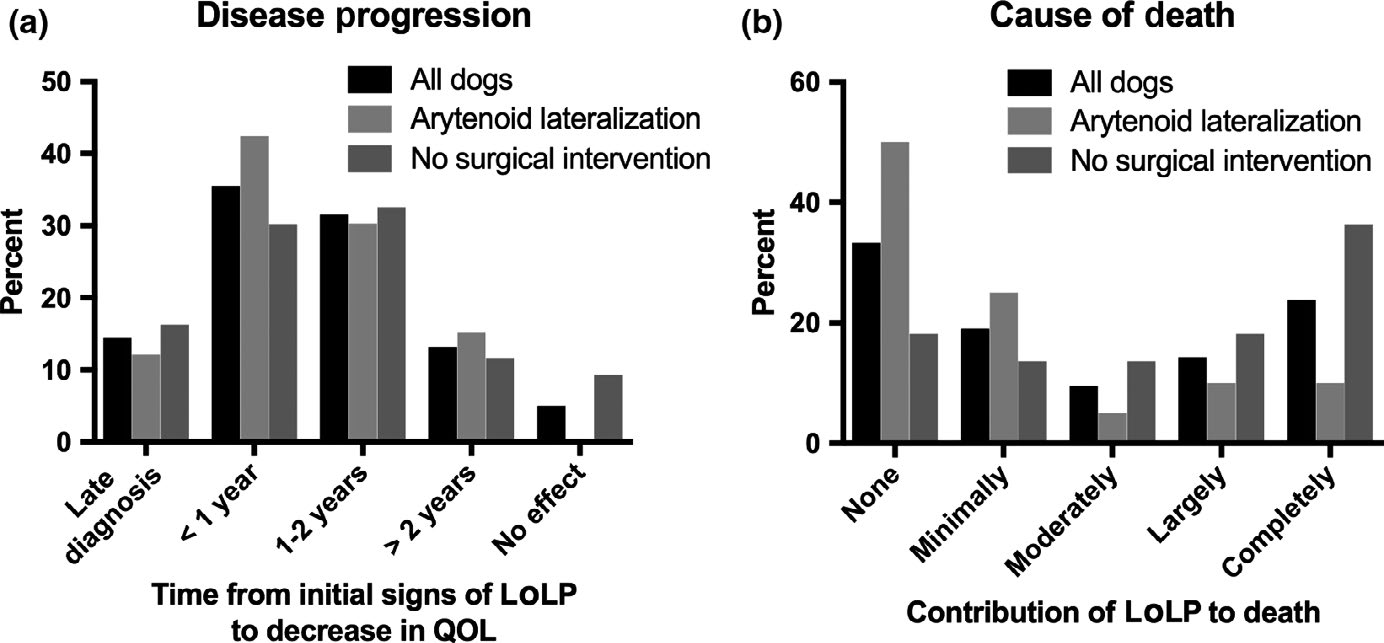
Owner perceived time from initial signs of late‐onset laryngeal paralysis (LoLP) to a decrease in their dog's quality of life (QOL), and owner perceived contribution of LoLP to their dog's death. (a) The time from which owners first noticed signs of LoLP to when they felt their dog's QOL was affected by the condition, a proxy for disease progression. The majority of owners felt that LoLP progressed to negatively impact their dog's QOL within 2 years of diagnosis. The response “no effect” refers to owners who did not feel LoLP affected their dog's QOL. “Late‐diagnosis” refers to responses from owners who did not know their dog had late‐onset laryngeal paralysis until the disease was severe. All dogs *n* = 76; Arytenoid lateralization *n* = 33; No surgical intervention *n* = 43. (b) The degree to which owners felt LoLP contributed to their dog's death is shown. Among all dog owners, 20 (47%) felt that LoLP was at least a large contributing factor to their dog's death. All dogs *n* = 42; Arytenoid lateralization *n* = 20; No surgical intervention *n* = 22. Results are presented as a percent of dogs from each group

Owners were asked how much time passed from when signs of LoLP were first evident to when LoLP progressed to the point that they felt their dog's QOL was affected, as a proxy to determine owner perception of disease progression. The most common response was <1 year (*n* = 27, 36%) (Figure 1a). The next most common response was between 1–2 years (*n* = 24, 32%). Ten owners (13%) felt LoLP progressed to affect QOL > 2 years after onset of clinical signs. Eleven owners (14%) did not know their dog had LoLP before the dog was severely affected. For this question, owners also were given the option of answering that they did not feel LoLP affected their dog's QOL; four owners (5%) selected this response. Overall, excluding owners who were not aware that their dog had LoLP until the animal was severely affected, 94% of owners felt that LoLP progressed to affect their dog's QOL. There was no significant difference in the amount of time from disease onset to LoLP effect on QOL between dogs that had unilateral arytenoid lateralization and dogs that did not (Table 1).

Regarding the degree to which owners felt LoLP contributed to their dog's cause of death or a decision to perform euthanasia (Figure 1b), 14 owners (33%) felt that LoLP did not contribute to their dog's death. Eight owners (19%) felt that while their dog died from another condition, LoLP did contribute somewhat to their dog's death. Four owners (9%) felt that LoLP was a large part of, but not the only reason for, their dog's death. Six owners (14%) felt that LoLP was the primary, but not only, factor that lead to their dog's death, whereas 10 owners (24%) felt that LoLP as the sole reason for their dog's death. Overall, 20 owners (47%) reported LoLP was, as a minimum, a large contributing factor to their dog's death. Owners whose dogs had unilateral arytenoid lateralization surgery felt that LoLP contributed less to their dog's cause of death than owners whose dogs did not have surgical intervention (*p* = .007) (median score 1.5 (range 1–5), median score 4 (range 1–5), respectively). A specific cause of death was able to be determined for 31 dogs from owner responses or pre‐necropsy medical records, if available (Table 2).

**Table 2:** Reported cause of death

Owner reported primary cause of death	Unilateral arytenoid lateralization	No surgical intervention
Complications associated with laryngeal paralysis	4	12
Cancer	5	4
Spinal degeneration or difficulty with mobility	2	1
Heart disease	1	0
Other neurological disease (acute tetraparesis, vestibular disease)	1	1
Unable to be determined	5	6

Note

A specific cause of death was able to be determined for 31 dogs from owner responses (*n* = 29) or pre‐necropsy medical records (*n* = 2), if available.

The median overall CHQLS score was 37 (range 20–45). The median score for happiness, out of a possible 12, was 12 (range 6–12). Median score for physical functioning, out of a possible 15, was 10 (range 5–15). Median score for hygiene, out of a possible 9, was 9 (range 5–9). Median score for mental status, out of a possible 9, was 7 (range 3–9). Owners of dogs that had unilateral arytenoid lateralization surgery scored their dogs median QOL significantly higher (*p* = .001) than owners of dogs that did not have surgical intervention (38.2 ± 3.8, 35.2 ± 5.6, respectively) (Table 1).

**Figure 2:**
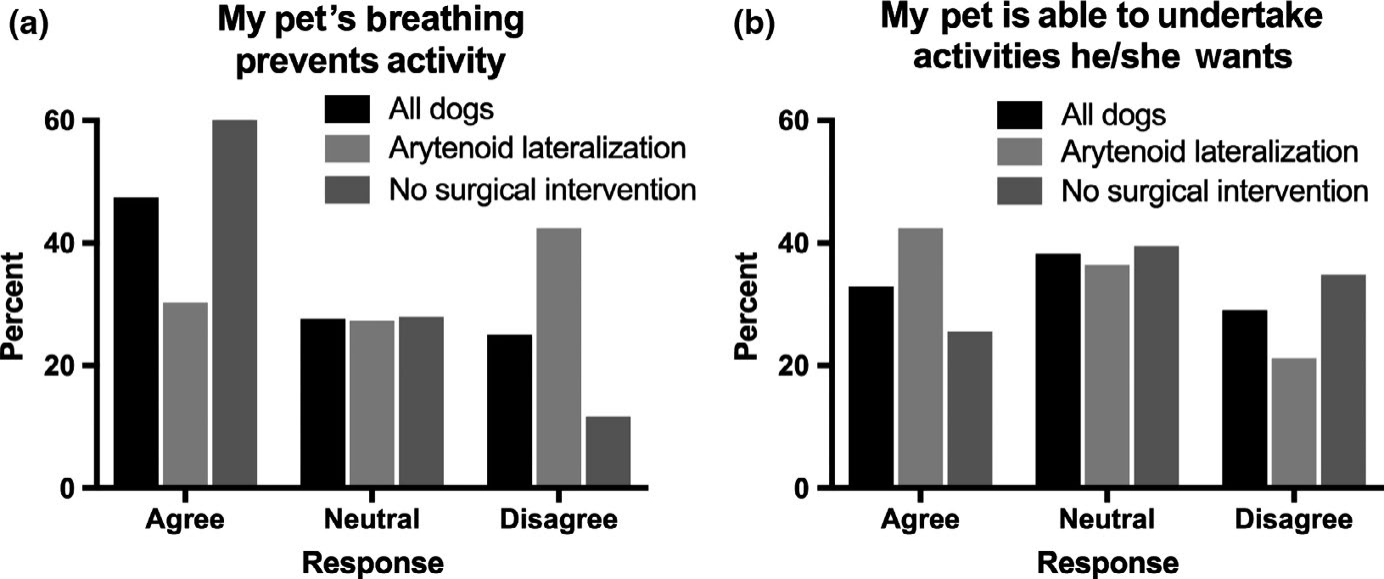
Owner responses to questions pertaining to their dog's activity with late‐onset laryngeal paralysis. (a) When owners were asked whether they agreed, were neutral or disagreed with the statement “My pet's breathing prevents activity,” nearly half of owners agreed. Owners of dogs that had unilateral arytenoid lateralization were more likely to respond that they did not feel that breathing affected their dog's activity, when compared with owners whose dogs did not have surgical intervention. (b) When owners were asked whether they agreed, were neutral or disagreed with the statement “My pet is able to undertake activities he/she wants,” the most common response was neutral, and no difference in responses were seen between groups. All dogs *n* = 76; Arytenoid lateralization *n* = 33; No surgical intervention *n* = 43. Results are presented as a percent of dogs from each group

When owners were asked whether they agreed, were neutral or disagreed with the statement that their pet's breathing prevented activity, 36 (47%) agreed, 21 (28%) were neutral and 19 (25%) disagreed. Responses from the 33 owners whose dogs had unilateral arytenoid lateralization were: 10 (30%) agreed, 9 (27%) were neutral and 14 (42%) disagreed. Responses from the 43 owners whose dogs did not have surgical intervention were as follows: 26 (60%) agreed, 12 (28%) were neutral and 5 (12%) disagreed. Owners of dogs that had unilateral arytenoid lateralization surgery were significantly more likely to think that breathing did not affect their pet's ability to undertake activity (*p* = .002) than owners of dogs that did not have surgical intervention (Figure 2a). When owners were asked whether they agreed, were neutral or disagreed with the statement that their pet was able to undertake activities he/she wanted, 25 (33%) agreed, 29 (38%) were neutral and 22 (29%) disagreed. Responses from the 33 owners whose dogs had unilateral arytenoid lateralization were as follows: 14 (42%) agreed, 12 (37%) were neutral and 7 (21%) disagreed. Responses from the 43 owners whose dogs did not have surgical intervention were as follows: 11 (26%) agreed, 17 (38%) were neutral and 15 (35%) disagreed. There was no difference in responses between owners of dogs who had unilateral arytenoid lateralization and owners of dogs that did not have surgical intervention (*p* = .10) (Figure 2b).

Of the 33 owners whose dogs underwent unilateral arytenoid lateralization, 32 reported that they felt the surgery positively affected their dog's QOL; one owner was unsure whether the surgery helped or not.

## 4 DISCUSSION

LoLP is a serious degenerative condition of older dogs (Stanley et al., 2010; Wilson & Monnet, 2016). This study was undertaken to establish owner perception of LoLP in the Labrador Retriever, which is the breed most commonly affected with LoLP. On the basis of these results, we accept our hypothesis that owners of Labrador Retrievers diagnosed with LoLP perceive the disease to be a life‐limiting condition that negatively affects their dog's QOL. Additionally, we found that owners who chose to have unilateral arytenoid lateralization surgery assessed their dogs to have a better QOL, and did not feel that LoLP contributed as much to their dog's cause of death compared with owners whose dogs did not have surgery. Importantly, of the 33 owners who elected to have arytenoid lateralization surgery, 32 were happy with this decision, whereas 1 owner was neutral.

The underlying cause of LoLP has not been determined, although the condition is accepted to be a component of a generalized polyneuropathy (Jeffery et al., 2006; Stanley et al., 2010). To minimize subject variance in this study, we chose to undertake this work using only owners of affected purebred Labrador Retrievers. The use of a specific breed was intentional to decrease subject variance, and therefore the results from this study may not be directly applicable across breeds. All dogs enrolled had signs of polyneuropathy in addition to laryngeal paralysis. Previous work has indicated that while not all dogs have clinical evidence of polyneuropathy at the time of LoLP presentation, many different breeds of dog develop signs of polyneuropathy within a year of presentation for LoLP (Stanley et al., 2010).

We used the CHQLS for this study (Lavan, 2013). While a number of owner surveys have been developed to evaluate QOL in veterinary medicine (Budke et al., 2008; Reid et al., 2013; Wiseman‐Orr, Scott, Reid, & Nolan, 2006), the CHQLS is specifically targeted to assess overall QOL in aging dogs, and has been validated in healthy older dogs (Lavan, 2013). The CHQLS survey is also brief and easy to complete, features which likely helped to encourage owner participation. To help maximize study enrolment, we collected questionnaire information by whatever means owners preferred, including over the telephone, online and by mail. Previous studies have shown that the method by which a questionnaire is obtained, such as in person versus online, can cause bias (Bowling, 2005). We found no correlation between the method by which the questionnaire was completed and owner responses (Edwards, 2010). Uniquely, a subset of owners took this survey after their dog's death; the CHQLS has not previously been used in this retrospective manner, and this is a limitation to this study. The Cronbach α coefficient for the survey indicated good internal consistency, suggesting that it was reasonable to add responses from the portion of owners that completed the survey after their dog's death with responses from owners whose dogs were alive at the time of the survey.

Owner perception of how LoLP affects their dog's QOL is important. LoLP is often considered by veterinarians to be a disease that has substantial impact on dogs and their owners. However, to optimize owner communication and recommendations, it is the perception of clients that must be understood. Furthermore, the impact of arytenoid lateralization on QOL, and whether it impacts perceived cause of death, has not been previously reported. To begin understanding owner perception of LoLP, we evaluated how quickly owners felt LoLP progressed to affect QOL, and how much owners felt LoLP contributed to their dog's death.

We asked owners how much time passed between when they noticed clinical signs of LoLP to when their dog's QOL was affected by LoLP. Most owners felt that the disease course affected their pet's QOL within 2 years of when they first noticed clinical signs. This is informative with regards to owner counselling when signs of LoLP are suspected, and may help guide when or if surgical intervention would be best for maximizing QOL during a pet's remaining years. While a majority of owners reported that LoLP affected their dog's QOL at some point, 14% of owners reported that they did not know their dog had LoLP until their pet was severely affected, highlighting the need for increased owner education regarding LoLP, particularity in commonly affected breeds such as the Labrador Retriever.

We also evaluated the degree to which owners perceived LoLP to contribute to their dog's death, as a proxy for understanding whether owners felt LoLP limited their dog's life‐span. Our results indicate that nearly half of owners viewed LoLP to be life‐limiting in their pet, as 48% of owners felt that LoLP was at least a large contributing factor in their dog's death. In contrast, 33% of owners reported that LoLP was not a factor contributing to their dog's death. While not all owners commented on their understanding of their dog's cause of death, owners who selected this response and provided a reason for their dog's death other than laryngeal paralysis listed a variety of reasons, with cancer being the most common (Table 2). For owners who did not report a specific cause of death, or stated that the cause of death would be in our institutional medical records, pre‐necropsy death reports were used if available. It should be noted that the degree to which LoLP contributed to death may be underestimated, as some owners felt their dog died of “spinal degeneration” or “mobility issues”, either of which could be a result of the underlying polyneuropathy associated with LoLP (Jeffery et al., 2006; Stanley et al., 2010). The prevalence of LoLP in Labradors is not known, and therefore the impact LoLP has on mortality in Labrador population as a whole cannot be extrapolated from this data.

We evaluated owner‐perceived age of disease onset. The average age of onset reported by owners was 10.4 years of age, which is consistent with previous studies (Milovancev et al., 2016; Snelling & Edwards, 2003; Wilson & Monnet, 2016). Owners who did not decide to have their dogs undergo airway opening surgery reported a later age of disease onset than owners who chose to have their dogs undergo unilateral arytenoid lateralization. It is possible owner interest in surgical intervention decreased with as pets got older. Alternatively, surgery may not have been recommended as strongly to owners of older dogs due to comorbidities or concerns for potential complications. The association between development of post‐operative complications and age remains unclear, with prior studies showing conflicting results (Bookbinder et al., 2016; MacPhail & Monnet, 2001; Wilson & Monnet, 2016). The true time of disease onset, rate of progression and severity are difficult to measure in client populations, but ultimately understanding how these factors relate to risks of post‐operative complications would be of substantial value. A more thorough evaluation of why owners did or did not choose surgery would be necessary to fully understand the connection between age of diagnosis and the decision whether to pursue airway opening surgery.

LoLP was significantly less likely to be perceived by owners to associate with cause of death in dogs that had unilateral arytenoid lateralization compared with those that did not, despite age of death not being statistically different between these groups. Interpretation of unblinded participant perception must be taken with caution. Owner perception of cause of death or QOL may be influenced by knowledge of whether their pet had surgical intervention, resulting in bias. This type of bias is not a concern when an outcome measure is unequivocal, such as age of death. Notably, while not statically significant, that age of death in dogs that had surgery was on average over 8 months later than dogs that did not have surgery. This observation supports prior work showing an increased risk of death in LoLP affected dogs that do not undergo a glottic opening procedure (Bookbinder et al., 2016). As maximizing QOL is a primary objective in veterinary medicine, age of death is arguably less important than QOL until death.

Dogs that underwent unilateral arytenoid lateralization had increased total CHQLS scores compared with dogs that did not have surgery. Owners of dogs that had unilateral arytenoid lateralization surgery were also less likely to report that breathing impacted their dog's activity. This overall positive perception of owners regarding the effect of unilateral arytenoid lateralization on their dog's QOL was further reflected in that 32 of 33 owners responded that they felt surgery positively affected their dog's QOL, with one owner not knowing whether the procedure of helpful as it had only recently been performed. Although follow‐up times were variable, this finding is similar to previous studies (Snelling & Edwards, 2003; White, 1989), which consistently report high levels of owner satisfaction with unilateral arytenoid lateralization. Despite this body of work, however, there is currently no standardized recommendation for when a glottic opening procedure should be considered in affected dogs. Recent work found that LoLP affected dogs with neurological comorbidities had increased risks of post‐operative complications (Bookbinder et al., 2016) (MacPhail & Monnet, 2001); presuming development of clinical evidence of polyneuropathy is a marker of disease progression (Stanley et al., 2010), this suggests that earlier surgical intervention may decrease risk of post‐operative complications. Taken together, based on client satisfaction and risk of post‐operative complications, surgical intervention should be considered as soon as LoLP has negative impacts on QOL. This is in contrast to common recommendations suggesting surgery should be delayed until a dog is experiencing episodes of cyanosis or severe respiratory distress. However, until a robust ability to quantify the relationship between disease severity and risk of post‐operative complications is available, timing of recommendations for intervention will likely remain ambiguous.

This study has a number of limitations. Results presented are based on owner feedback, and must be viewed within this intentional limitation. It is likely that owners had differing abilities to identify mild early changes that could be associated with the LoLP, and differing perspectives regarding when and how the disease affected their dogs. Detection of clinical signs is also likely influenced by factors such as overall body condition, general activity level, or anatomic variations in resting glottic area. LoLP is most commonly part of a degenerative polyneuropathy that affects mobility, oesophageal function and breathing (Stanley et al., 2010). Therefore, changes in QOL due to decreased mobility associated with LoLP may be misinterpreted by owners, thus underestimating the true impact of LoLP on QOL. The inclusion of an unaffected control group should be considered for a future prospective study, as this would enable age‐related changes in QOL to be accounted for in the analysis. This is the first time CHQLS has been used in a clinical study of diseased dogs, and this questionnaire has not been validated for this purpose. Information was used from owners of dogs that were deceased, which could result in bias. Complete medical records were not available for most dogs in this study, and therefore details beyond the type of glottic opening surgery could not be included in our analysis. As such, specific details regarding comorbidities and true cause of death were considered beyond the scope of this study. As QOL was not accessed before and after surgery for dogs that underwent unilateral arytenoid lateralization, it is not possible to determine whether QOL scores were higher in group because of surgery, or if surgery was elected in dogs with better QOL. Additionally, it is important that conclusions regarding the potential benefit of unilateral arytenoid lateralization be taken in light of a potential owner placebo effect (Conzemius & Evans, 2012). Further investigation to quantify changes in dog activity levels before and after surgery should be considered to help clarify potential benefits of glottic opening procedures (Piazza, Csomos, Sample, & Hardie, 2018).

## 5 CONCLUSIONS

This work is the first to document owner perception that LoLP is a life‐limiting disease that has a negative impact on pet QOL. We found dogs that underwent unilateral arytenoid lateralization had higher QOL scores than dogs that did not have surgical intervention, and were less likely to have LoLP perceived as a large contributor towards death. This information is helpful when discussing expectations and surgical options with owners of Labrador Retrievers diagnosed with LoLP.

## CONFLICT OF INTEREST

No conflicts of interest have been declared.
