# Supplementary material for: Late‐onset laryngeal paralysis: Owner perception of quality of life and cause of death
Source: Vet Med Sci. 2020 Jan 25;6(3):306–13. doi: 10.1002/vms3.240 (PMC7397904; doi:10.1002/vms3.240)
Supplement: Supplementary file 1 [file VMS3-6-306-s001.docx]

Your Name: __________________ Dog’s Name: _______________ Today’s Date: _________

Date of birth: _________________

**Is your dog alive?** Yes No (if No, date of death: ________________)

**How old was your dog when you noticed signs you attribute to laryngeal paralysis?**  _________________

**How quickly from when you first noticed signs of laryngeal paralysis did the symptoms progress to the point you felt your dog’s quality of life was affected**? (one answer)

- I don’t think my dog’s quality of life is/was affected by laryngeal paralysis
- Over more than 2 years
- Over 1-2 years
- Over less than one year
- I didn’t know my dog had laryngeal paralysis until he/she was very affected

**Did your dog have airway surgery to address laryngeal paralysis**? (circle one) Yes No

**If yes**, do you feel surgery positively affected your dog’s quality of life? (circle one) Yes No

**If your dog is deceased, how much of a role do you feel the consequences of laryngeal paralysis contributed to your dog’s death/the decision to perform humane euthanasia?**

- None: Laryngeal paralysis was not a contributing factor with regards to my dog’s death
- Minimally: I feel my dog died of another condition, but laryngeal paralysis did contribute somewhat to his/her death
- Moderately: Laryngeal paralysis was large part of, but not the only reason for, my dog’s death
- Largely: Laryngeal paralysis was the primary factor, but not the only factor, that lead to my dog’s death
- Completely: I believe laryngeal paralysis, or the consequences of laryngeal paralysis, was the sole reason for my dog’s death.

**Please list any other conditions your dog has been diagnosed with over his/her life, and the approximate age of that diagnosis:**

| Condition | Age of Diagnosis |
| --- | --- |
|  |  |
|  |  |
|  |  |

**Comments/Other information you feel are important**: ___________________________________________

**Lifestyle and Quality of Life Assessment**

If your dog is alive, please respond with regards to his/her current status. If your dog has died, please respond with regards to his/her status as you remember how laryngeal paralysis affected his/her life. We will analysis this data with respect to other medical conditions you have listed above.

***Please circle one: 1 (Disagree) 2 (Neutral) 3 (Agree)***

| Happiness | | | |
| --- | --- | --- | --- |
| My pet wants to play | 1 | 2 | 3 |
| My pet responds to my presence | 1 | 2 | 3 |
| My pet enjoys life | 1 | 2 | 3 |
| My pet has more good days than bad days | 1 | 2 | 3 |
| My pet is able to undertake activities he/she wants | 1 | 2 | 3 |
| Physical functioning | | | |
| My pet sleeps more, is less awake | 1 | 2 | 3 |
| My pet is in pain | 1 | 2 | 3 |
| My pet moves normally | 1 | 2 | 3 |
| My pet lays in one place all day long | 1 | 2 | 3 |
| My pet is as active as he/she has been | 1 | 2 | 3 |
| My pet’s breathing prevents activity | 1 | 2 | 3 |
| Hygiene | | | |
| My pet keeps him/herself clean | 1 | 2 | 3 |
| My pet smells like urine or has skin irritation | 1 | 2 | 3 |
| My pet’s hair is greasy, matted, rough looking | 1 | 2 | 3 |
| Mental status | | | |
| My pet seems dull or depressed, not alert | 1 | 2 | 3 |
| My pet pants frequently, even at rest | 1 | 2 | 3 |
| My pet shakes or trembles occasionally | 1 | 2 | 3 |
